# Olive Leaf Extract Attenuates Inflammatory Activation and DNA Damage in Human Arterial Endothelial Cells

**DOI:** 10.3389/fcvm.2019.00056

**Published:** 2019-05-16

**Authors:** Blaž Burja, Tadeja Kuret, Tea Janko, Dijana Topalović, Lada Živković, Katjuša Mrak-Poljšak, Biljana Spremo-Potparević, Polona Žigon, Oliver Distler, Saša Čučnik, Snezna Sodin-Semrl, Katja Lakota, Mojca Frank-Bertoncelj

**Affiliations:** ^1^Department of Rheumatology, University Medical Centre, Ljubljana, Slovenia; ^2^Department of Rheumatology, Center of Experimental Rheumatology, University Hospital Zurich, Zurich, Switzerland; ^3^Chair of Clinical Biochemistry, Faculty of Pharmacy, University of Ljubljana, Ljubljana, Slovenia; ^4^Faculty of Mathematics, Natural Science and Information Technology, University of Primorska, Koper, Slovenia; ^5^Department of Pathobiology, Faculty of Pharmacy, University of Belgrade, Belgrade, Serbia

**Keywords:** OLE, SAA, HCAEC, inflammation, atherosclerosis, microRNA, DNA damage

## Abstract

Olive leaf extract (OLE) is used in traditional medicine as a food supplement and as an over-the-counter drug for a variety of its effects, including anti-inflammatory and anti-atherosclerotic ones. Mechanisms through which OLE could modulate these pathways in human vasculature remain largely unknown. Serum amyloid A (SAA) plays a causal role in atherosclerosis and cardiovascular diseases and induces pro-inflammatory and pro-adhesive responses in human coronary artery endothelial cells (HCAEC). Within this study we explored whether OLE can attenuate SAA-driven responses in HCAEC. HCAEC were treated with SAA (1,000 nM) and/or OLE (0.5 and 1 mg/ml). The expression of adhesion molecules VCAM-1 and E-selectin, matrix metalloproteinases (MMP2 and MMP9) and microRNA 146a, let-7e, and let-7g (involved in the regulation of inflammation) was determined by qPCR. The amount of secreted IL-6, IL-8, MIF, and GRO-α in cell culture supernatants was quantified by ELISA. Phosphorylation of NF-κB was assessed by Western blot and DNA damage was measured using the COMET assay. OLE decreased significantly released protein levels of IL-6 and IL-8, as well as mRNA expression of E-selectin in SAA-stimulated HCAEC and reduced MMP2 levels in unstimulated cells. Phosphorylation of NF-κB (p65) was upregulated in the presence of SAA, with OLE significantly attenuating this SAA-induced effect. OLE stabilized SAA-induced upregulation of microRNA-146a and let-7e in HCAEC, suggesting that OLE could fine-tune the SAA-driven activity of NF-κB by changing the microRNA networks in HCAEC. SAA induced DNA damage and worsened the oxidative DNA damage in HCAEC, whereas OLE protected HCAEC from SAA- and H_2_O_2_-driven DNA damage. OLE significantly attenuated certain pro-inflammatory and pro-adhesive responses and decreased DNA damage in HCAEC upon stimulation with SAA. The reversal of SAA-driven endothelial activation by OLE might contribute to its anti-inflammatory and anti-atherogenic effects in HCAEC.

## Introduction

Cardiovascular diseases, including coronary artery disease, are the leading cause of death globally, with atherosclerosis being the most common underlying reason for cardiovascular morbidity and mortality. In addition, systemic inflammation (present in a number of chronic diseases, including metabolic syndrome and rheumatoid arthritis) drives accelerated atherosclerosis (1) and patients with these diseases are at risk for developing atherosclerosis and its complications. Serum amyloid A (SAA), a major acute phase protein, is increased in chronic inflammatory diseases and coronary artery disease (2, 3). SAA was shown to be an early causal agent for atherosclerosis in animal models (4, 5). Serum levels of SAA have been reported to correlate with severity of atherosclerosis (6, 7) and have been utilized as a predictor of mortality following acute myocardial infarction (8, 9). In addition, SAA was shown to enhance monocyte and lymphocyte recruitment, directly stimulating foam cell formation, associating with HDL/LDL particles and compromising reverse cholesterol transport, among other processes (7). Furthermore, SAA was reported to induce matrix metalloproteinases (MMP), as well as increase biglycan synthesis and influence retention of lipoproteins by vascular proteoglycans (10). Early on, Ridker reported that even chronic low grade inflammation may lead to atherosclerosis and the development of cardiovascular diseases (11), emphasizing the importance of measuring systemic inflammatory markers, such as SAA.

Atherosclerotic lesions typically develop in coronary arteries and the activation of endothelium represents one of the crucial early steps in the development of atherosclerotic lesions in the coronary arterial system. Human coronary artery endothelial cells (HCAEC) therefore represent a suitable *in vitro* model to study the mechanisms of endothelial activation and atherosclerosis. HCAEC strongly respond to stimulation with SAA, activating a number of pro-inflammatory, pro-adhesive and pro-coagulant responses, as measured by increased expression of interleukin 6 (IL-6), interleukin 8 (IL-8), vascular cell adhesion molecule (VCAM1) and tissue factor (12). Elevated amounts of systemic SAA in patients with coronary artery disease may represent a potential pathophysiological link between inflammation, lipoprotein metabolism, oxidative stress and the development of atherosclerosis (2). DNA damage is emerging as a crucial player in accelerating the development and progression of atherosclerosis. In addition to microRNAs, which are important regulators of cellular processes leading to atherosclerosis (13–15), DNA damage has been reported to be a biomarker of atherosclerosis in monocytes (16). There is a lack of information concerning whether DNA damage and alterations in microRNA networks accompany or contribute to the SAA-driven endothelial activation.

Olive oil represents the basis of atheroprotective effects of the Mediterranean diet, and olive leaf extract (OLE) contains a much higher concentration of active polyphenol compounds compared with extra virgin olive oil (17). OLE from the olive tree (*Olea europaea* L.) is used as a food supplement or as an over-the-counter drug for a variety of benefits including its anti-arrhythmic, anti-atherosclerotic (18), anti-hypertensive, antioxidant, anti-tumor, anti-proliferative, anti-inflammatory (19, 20), and anti-fibrotic (20, 21) effects. The 80% alcoholic extract of olive leaves has also been included in the European Pharmacopeia (Ph. Eur.) based on a subset of its protective properties (20). The polyphenols of the olive tree activated major anti-oxidant pathways, including transcription factor Nrf2, as well as AMPK, IGF/Akt, and mTOR, thereby acting similarly to caloric restriction mimickers (20).

Dry OLE is rich in water-soluble phenolic compounds, specifically secoiridoid oleuropein (17%), whereas the remaining compounds [apigenine-7-O-glucoside, luteolin-7-O-glucoside, quercetin, and caffeic acid] are present in substantially smaller amounts [<0.1%] (22). Secoiridoid oleuropein consists of a polyphenolic molecule known as hydroxytyrosol, bound to elenolic acid and a glucose molecule. OLE and its compounds can repress the expression of a number of pro-inflammatory genes. Specifically, oleuropein decreased the expression of IL-6 and IL-1β in colon during DSS-induced colitis (23) and in LPS-stimulated macrophages (Raw264.7) (24). Peripheral blood mononuclear cells (PBMC) from healthy individuals, who consumed OLE on a single occasion, expressed less IL-8 when stimulated *ex vivo* with LPS (25), and serum IL-8 levels decreased in hypertensive individuals who consumed OLE for 6 weeks (26). IL-8 was also one of the most downregulated genes in PBMC from healthy subjects who consumed OLE for 8 weeks. The major contributors to these effects were downregulation of the arachidonic acid and NF-κB pathways by OLE (27). The inflammatory molecules MCP-1, VCAM-1, and TNF-α were down-regulated in thoracic aorta of rabbits on a high lipid diet supplemented with OLE, as compared to a high lipid diet alone (18). Oleuropein and hydroxytyrosol, the constituents of OLE, inhibited the expression of both VCAM-1 and ICAM-1 in human umbilical vein endothelial cells, further accompanied by a decreased activation of NF-κB and AP-1 and reduced monocyte adhesion (28). These studies suggest that OLE could affect pro-inflammatory and pro-adhesive cellular responses during the atherosclerotic process in the arterial wall.

In our present work, we show that OLE significantly attenuated the SAA-driven pro-inflammatory and pro-adhesive responses of HCAEC and modulated SAA-induced miR-146a and let-7e. OLE decreased SAA-induced phosphorylation of NF-κB and protected endothelial cells from SAA-induced DNA damage. This suggests that OLE might exhibit multiple athero-protective effects in coronary arterial vasculature.

## Materials and Methods

### Materials

Lyophilized human recombinant SAA (Peprotech EC Ltd., London, UK) was reconstituted according to manufacturer's instructions to stock concentration and stored until usage at −20 or −80°C. The final concentration of SAA in experiments was 1,000 nM. Dry OLE EFLA®943 (Frutarom Switzerland Ltd., Wadenswil, Switzerland) was originally manufactured by applying an ethanol extraction procedure (80% m/m) from the dried leaves of olive tree, standardized to 16–24% of oleuropein. Dry OLE powder was diluted in phosphate buffered saline (PBS) to a final concentration of 0.1 g/ml, unless otherwise indicated.

### Cell Culture

HCAEC from four different donors were purchased from Lonza (Walkesville, Maryland, USA). The cells were plated onto 25 cm^2^ flasks and 6 or 24 well-plates (TPP, Trasadigen, Switzerland) and cultured at 37°C in a humidified atmosphere at 5% CO_2_. HCAEC were grown in EGM-2M medium (CambrexBioScience, Walkesville, Maryland, USA) containing 5% fetal bovine serum. For experiments, subconfluent cell cultures were used between passages 4 and 6 in serum-free medium, with pretreatment of OLE for 45 min, followed by addition of SAA for 24 h, unless otherwise indicated. Prior to experiments, cells were incubated in serum-free medium for 2 h.

### RNA Isolation and Reverse Transcription Polymerase Chain Reaction Analysis

Prior to reverse transcription polymerase chain reaction (RT-PCR), total RNA from endothelial cell cultures was isolated using Total RNA Isolation System RNeasyPlus Micro (Qiagen, Germany), following manufacturer's instructions. The purity and amount of RNA were determined by measuring the OD at a ratio of 260 to 280 nm. One microgram of total RNA was transcribed into cDNA by Reverse Transcription System (Promega, Madison, WI, USA). For microRNA measurements, 5 ng of total RNA was reverse-transcribed using the TaqMan®MicroRNA Reverse Transcription Kit and miRNA-specific RT primers (Applied Biosystems).

### Real Time PCR

The expression of mRNA was measured with real time PCR ABI Step One (Applied Biosystems, Foster City, CA, USA) using Kapa Sybr master mix (Sigma-Aldrich, Germany), forward and reverse primers (200 nM each) and 10 ng cDNA per well. Primer sequences and conditions for RT-PCR are indicated for each primer set in Supplementary Table 1. Dissociation curves showed one peak in each PCR reaction. All experiments were performed in triplicates. GAPDH was used as an endogenous normalization control. The gene expression results were calculated with the 2^CtΔΔ^ method. The expression of microRNAs was determined with real-time PCR (7900HT Fast real-time PCR system) using TaqMan®probes and TaqMan Universal PCR Master Mix (all Applied Biosystems, Life Technologies Foster City, CA, USA). RNU48 was used as an endogenous control. Differences in microRNA expression were calculated with the 2^CtΔΔ^ method.

### Western Blot

Whole cell lysates were prepared from HCAEC using RIPA buffer containing Halt protease inhibitors (Pierce, Rockford, IL, USA) and phosphatase inhibitors (Cayman Chemical, Ann Arbor, MI, USA). The concentration of proteins was determined with the Bradford assay and equal amounts of proteins were loaded per gel pocket. α-tubulin was used as a loading control. Whole cell lysates, mixed with loading buffer, were separated on 10% SDS polyacrylamide gels and electroblotted onto nitrocellulose membranes (Whatman). Membranes were blocked for 1 h in 5 % (w/v) non-fat milk in TBS-T. After blocking, the membranes were probed with rabbit anti-phospho NF-κB p65 and rabbit anti-NF-κB p65 (S536 and E498, respectively, both at dilutions of 1:1000 Cell Signaling Technology, Danvers, MA, USA). As secondary antibodies, horseradish peroxidase-conjugated goat anti-rabbit (Cell Signaling Technology, Danvers, MA, USA) were used. Signals were detected using Femto Luminol (ThermoFisher Scientific, Hempstead, UK) with G:Box (Syngene, Cambridge,UK). Densitometry analysis of protein bands was carried out using the Fusion FX software (Vilber Lourmat). For quantification of Western blots, the levels of phosphorylated NF-κB were normalized to the levels of total NF-κB.

### Protein Detection by ELISA

Supernatants from untreated and treated HCAEC were spun down (300 g, 5 min) and cell-free supernatants frozen at −80°C until usage. The assays were performed using commercial ELISA kits and were done in duplicates, according to the manufacturers‘ instructions. IL-6 and IL-8 ELISA kits were purchased from Invitrogen (Frederick, MD, USA), while GROa and MIF quantikine ELISA were from R&D Systems (Minneapolis, USA). Absorbance was measured at 450 nm with the microplate absorbance reader (Tecan, Groening, Austria). The concentrations of analytes were calculated from standard curves and multiplied by the dilution factor.

### Comet Assay

**Scheme 1:**
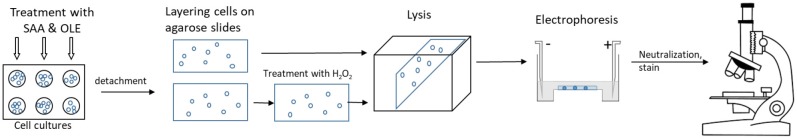
Scheme of COMET Analysis.

HCAEC were grown in 6 well-plates. After treatment with SAA ± OLE, cells were detached using Acutasse (Sigma Aldrich) and spun down (5 min, 300 g). COMET was performed as previously described (29). Briefly, cells were resuspended in 0.067% low melting agarose (Sigma Aldrich) at 37^0^C and were layered onto slides using a coverslip. Cells from each 6 well were layered onto 4 slides and after cooling at 4^0^C for 10 min, coverslips were removed. Two slides were treated with 3% H_2_O_2_ at 37°C and rinsed with PBS. The remaining two slides were immediately covered with an additional layer of 0.05% low melting agarose and were placed (after cooling) into fresh alkaline lysis buffer overnight. The lysis buffer was prepared from 89 ml of stock solution containing 2.5M NaCl, 100 mM EDTA, 10 mM Tris-base at pH 10, 10 ml DMSO and 1 ml Triton-X. Slides were then immersed into cold, alkaline electrophoresis buffer (300 mM NaOH, 1 mM EDTA freshly prepared from stock 10 M NaOH and stock 200 mM EDTA, pH 10) for 30 min, after which electrophoresis was run (30 min, 25 V, 300 mA). This was followed by 2 repeated rinses for 10 min with neutralization buffer (0.32 M Tris base at pH 7.5) and water. Slides were stained with Sybr Green I, and 100 cells per slide were scored under the fluorescence microscope (Nikon eclipse TE 300 and AxioImager Z1, Carl Zeiss, 400x magnification). The average of the duplicate slides was calculated and three biological replicates were performed per experimental condition (Scheme 1).

### Statistical Analysis

Data was presented as mean ± standard deviation (SD), unless otherwise indicated. The differences between the various treated and control groups were analyzed with RM one way ANOVA test with Tukey's method for multiple comparisons. All data was analyzed with the GraphPad Prism 7.0 software. A difference of *p* < 0.05 was considered statistically significant. DIANA-miRPath v3.0 software was used for assessment of miRNA regulatory roles and the identification of controlled pathways (30).

## Results

### OLE Reduces the SAA-Driven Release of Pro-Inflammatory Cytokines and Chemokines From HCAEC

**Figure 1:**
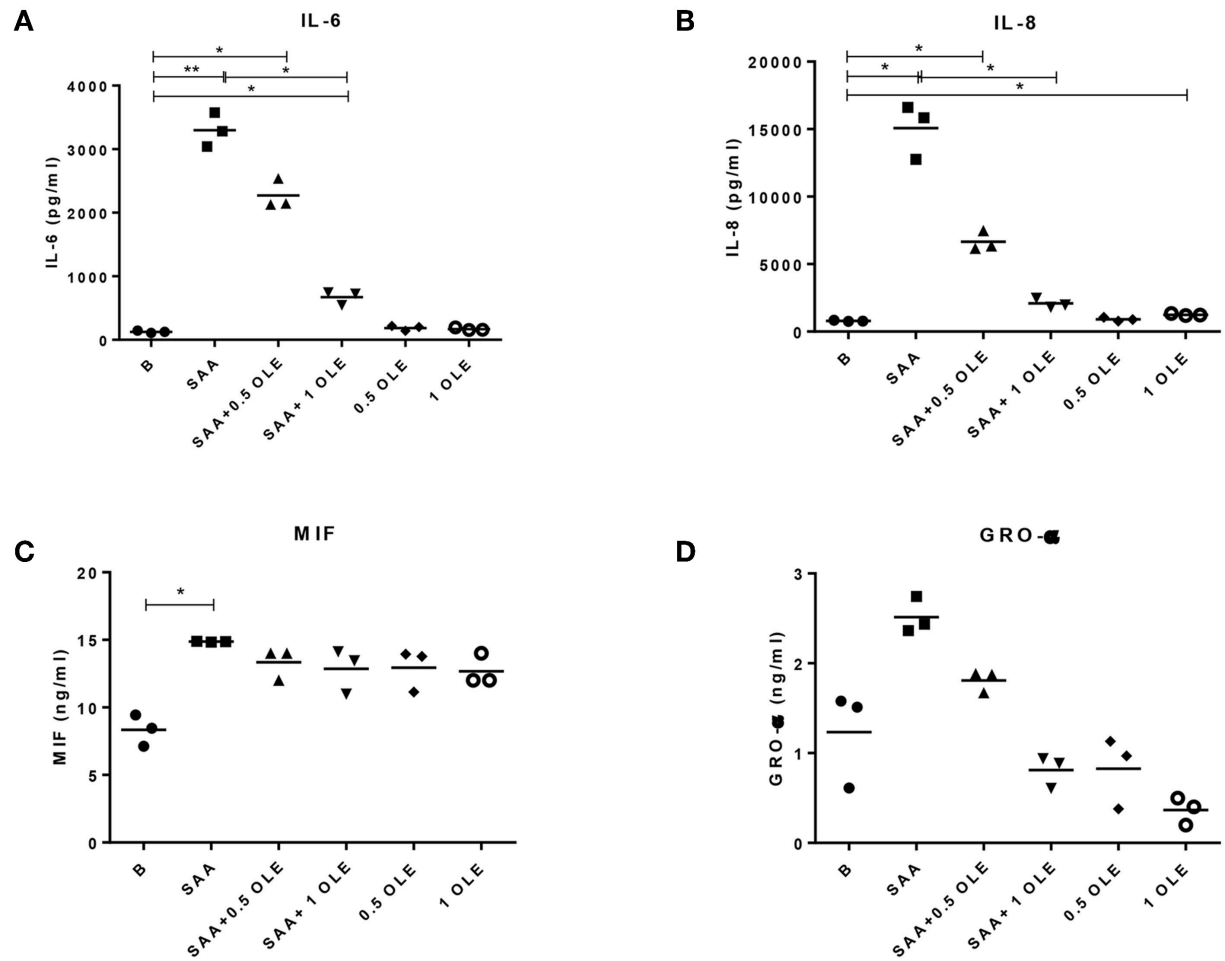
OLE decreases SAA-driven release of pro-inflammatory cytokine IL-6 and chemokine IL-8 from HCAEC. The amount of IL-6 **(A)**, IL-8 **(B)**, MIF **(C)**, and GRO-α **(D)** in cell culture supernatants were measured by ELISA. The mean of 3 biological replicates is shown. HCAEC were treated with OLE (0.5 mg/ml, 1 mg/ml) 45 min prior to SAA addition (1,000 nM, 24 h). Data was analyzed using RM one-way ANOVA test with Tukey's multiple comparison test *p > 0.05 **p > 0.01.

Treatment of HCAEC with OLE alone or in combination with SAA did not decrease the viability of HCAEC when compared to untreated cells (data not show). Inflammatory chemokines and cytokines play an important role in recruiting inflammatory cells and sustaining inflammation in chronic inflammatory diseases. To mimic the inflammatory milieu in the vasculature of patients with an activated systemic inflammatory response, we stimulated HCAEC with SAA (1000 nM or 12 mg/l). While high concentrations of SAA are commonly reached in the circulation of patients with infections, inflammatory and autoimmune diseases or trauma (31), chronically elevated low levels of SAA can already cause atherosclerosis (11). Stimulation of HCAEC with SAA significantly increased the release of IL-6, IL-8, and MIF (Figures 1A,B). Treatment with OLE reduced this SAA-induced release of IL-6 and IL-8 in a dose-dependent manner (Figures 1A,B). While 1 mg/ml OLE significantly decreased the release of IL-6 into supernatants of the SAA-stimulated HCAEC, the release of IL-8 returned to baseline levels. Additionally, similar effects of OLE were observed on the SAA-driven release of GROα, although significant changes were not observed (most probably due to large differences in the basal production of GROα in HCAEC). SAA-driven release of MIF was not affected (Figures 1C,D). Overall, this suggests that OLE can modulate the expression of a subset of the SAA-induced pro-inflammatory genes.

### OLE Alters the mRNA Expression of SAA-Induced E-Selectin in HCAEC

**Figure 2:**
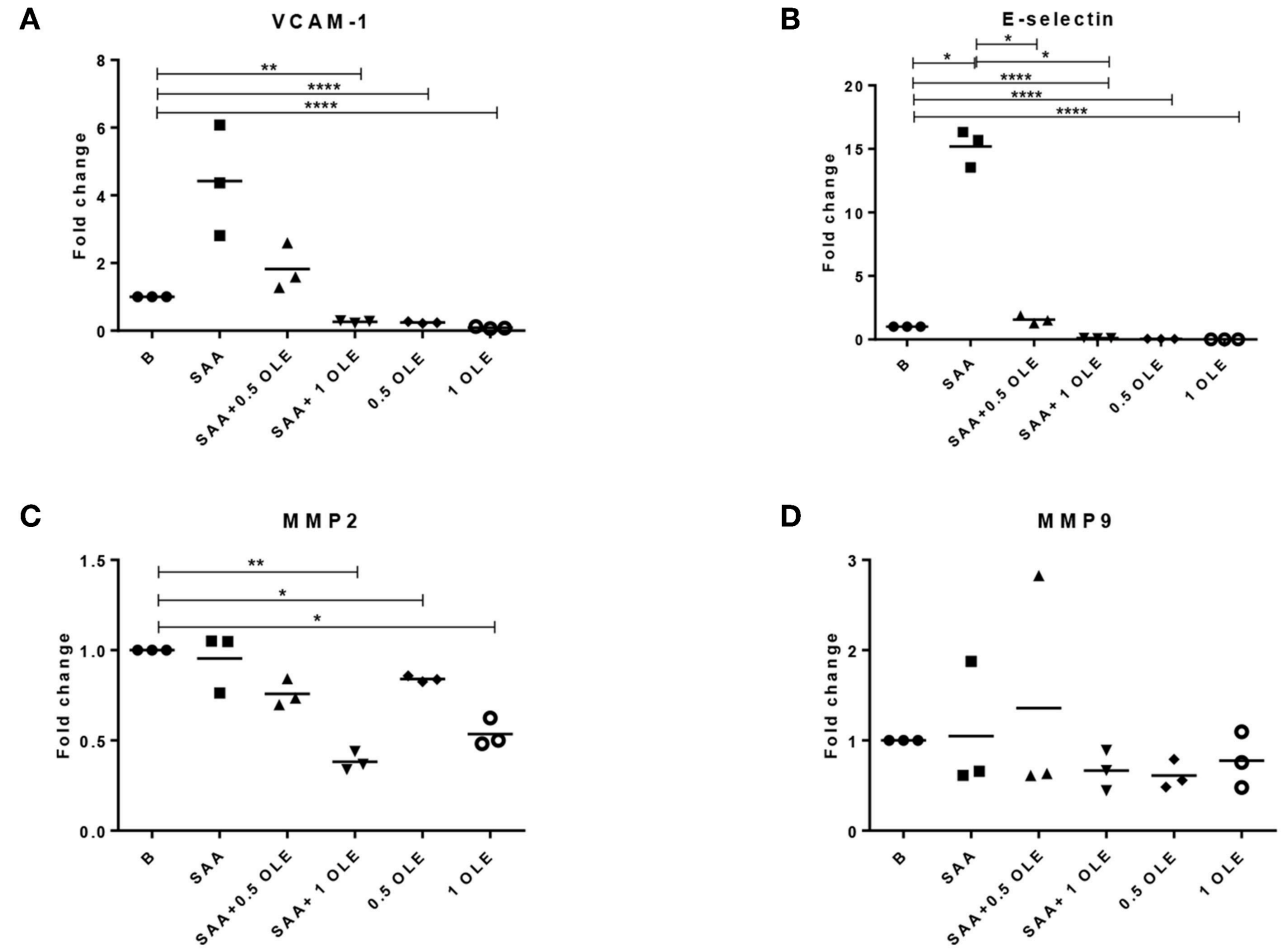
OLE decreases mRNA expression of adhesion molecules and MMP2 in HCAEC. The expression of VCAM-1 **(A)**, E-selectin **(B)**, MMP2 **(C)**, and MMP9 **(D)** mRNA was determined by qPCR. Mean of 3 biological replicates is shown. HCAEC were treated with OLE (0.5 mg/ml, 1 mg/ml) 45 min prior to SAA addition (1,000 nM, 24 h). Data was analyzed using RM one way ANOVA test with Tukey's multiple comparison test *p > 0.05 **p > 0.01; ****P < 0.0001.

SAA significantly increased the expression of E-selectin mRNA in HCAEC, whereas VCAM-1 mRNA was up-regulated on average 4-fold above baseline with a notable variation between the donors (significance not reached) (Figures 2A,B). SAA-driven upregulation of E-selectin mRNA returned to baseline in the presence of OLE (Figure 2B). Moreover, OLE significantly down-regulated the constitutive expression of both E-selectin and VCAM-1 mRNAs (Figures 2A,B). While SAA strongly enhanced pro-inflammatory and pro-adhesive activities in HCAEC, it did not alter significantly the expression of MMP2, MMP9 and mRNAs (Figures 2C,D).

### SAA-Induced NF-κB Phosphorylation Is Reduced by OLE

**Figure 3:**
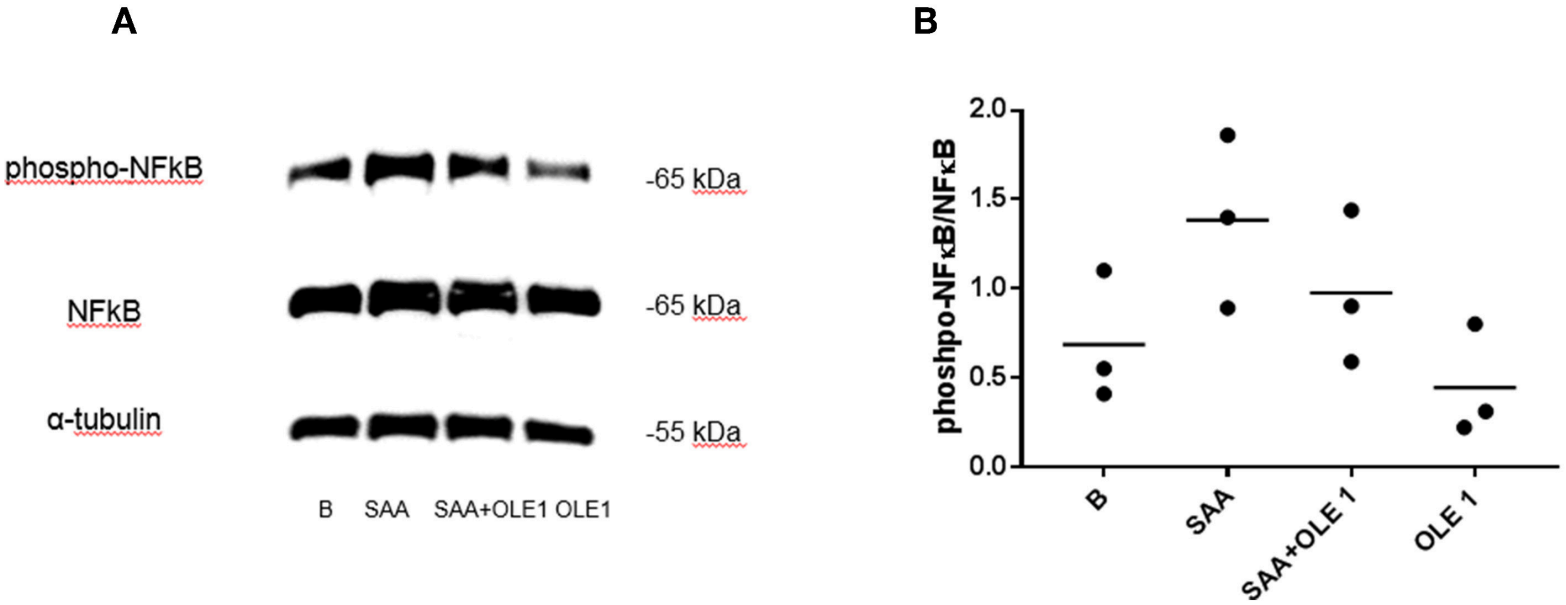
OLE attenuates SAA-induced phosphorylation of NF-κB. **(A)** Western blot shows phosphorylation of NF-κB in HCAEC pretreated with OLE for 1 h and then treated with OLE ± SAA for 1 h (SAA 1,000 nM, OLE 1 mg/ml). Representative blot of *n* = 3 biological replicates. **(B)** Densitometry analysis of protein bands was carried out using the Fusion FX software (Vilber Lourmat), *n* = 3 biological replicates. For quantification of Western blots, the levels of phosphorylated NF-κB were normalized to the levels of total NF-κB. Equal amounts of protein were loaded per gel pocket. α-tubulin was used as a loading control.

The SAA-induced activation of gene expression coincided with the increase in the phosphorylation of NF-κB p65 (Figure 3). NF-κB is the central transcription factor in the expression of pro-inflammatory genes including those coding for cytokines, chemokines, and adhesion molecules (32). The SAA-increased phosphorylation of NF-κB was decreased in the presence of OLE (Figure 3). This shows that OLE could exert its anti-inflammatory and anti-adhesive effect also through modulation of NF-κB signaling.

### OLE Protects HCAEC From SAA-Induced and H_2_O_2_-Induced DNA Damage

**Figure 4:**
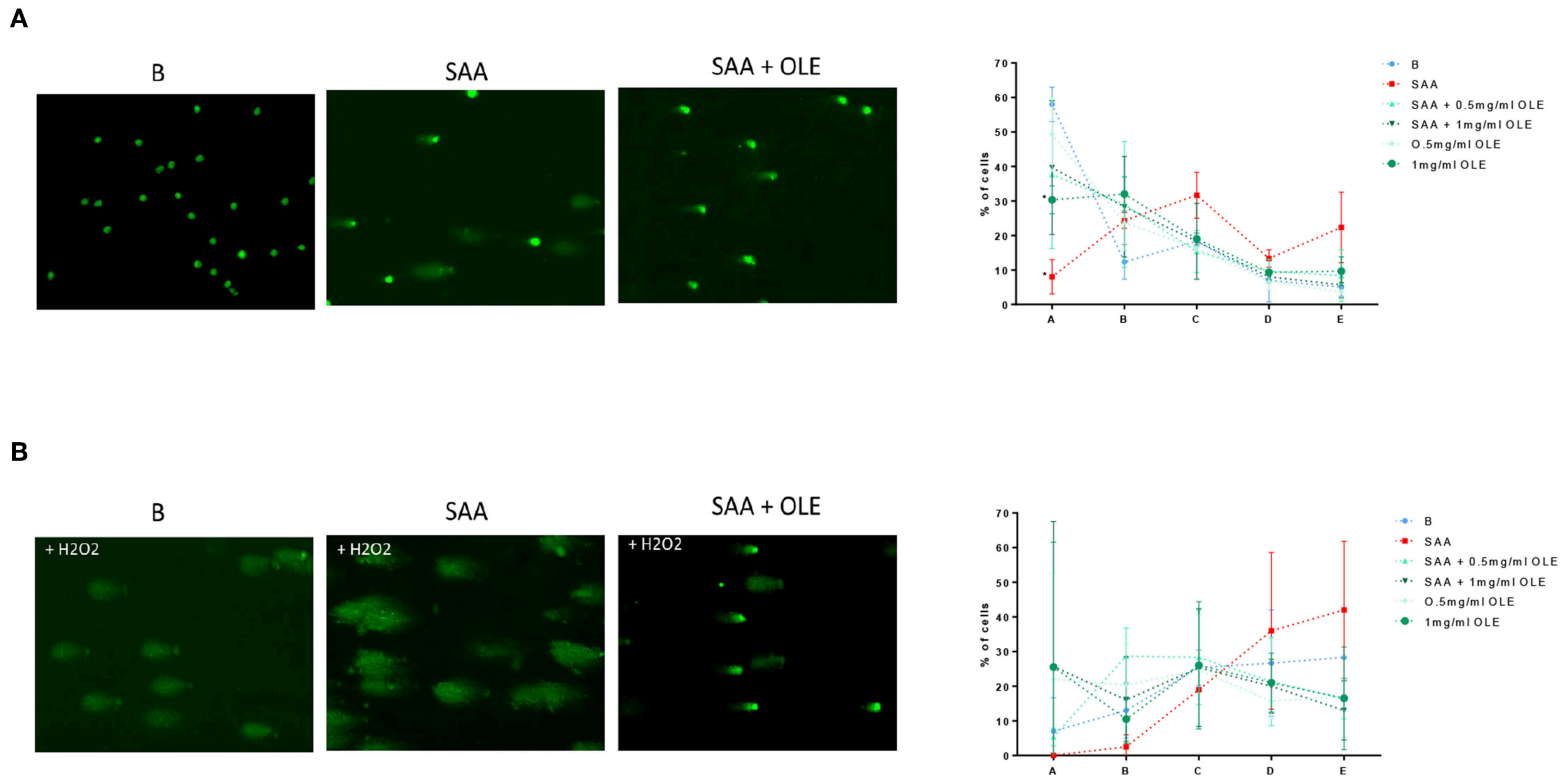
OLE attenuates SAA-induced and H_2_O_2_-induced DNA damage in HCAEC. COMET assay was performed without **(A)** and with oxidative treatment (3% H_2_O_2_) **(B)**. Average from 3 biological replicates of the COMET assay for each experimental condition was performed on duplicate slides, 100 cells were evaluated per slide according to the following categories: A - no tail (no DNA damage), B - slight tail, C - strong signal in tail, but also in nucleus, D - majority of DNA in tail, E - no DNA in round nucleus.

Oxidative stress occurs when the formation of free radicals increases or when the antioxidant capacity of a cell is decreased, leading to DNA damage (33). COMET assay is a sensitive method to measure DNA damage in cells (34), where the relative amount of DNA in the COMET tail indicates the frequency of DNA breaks. Our results demonstrated that under basal conditions, on average 58% of HCAEC exhibited no COMET tails (condition A), whereas only 8% of the SAA-treated HCAEC were without COMET tails (Figure 4A). While 0.5 mg/ml OLE largely mimicked the background control in category A, a smaller percentage of cells, around 30%, exhibited no COMET tails when treated with 1 mg/ml OLE (Figure 4A). Nevertheless, the treatment with 0.5 mg/ml and 1 mg/ml OLE decreased DNA damage in the SAA-treated HCAEC, with 38% and 40% of HCAEC, respectively, exhibiting no DNA damage (no COMET tails) (Figure 4A). This data shows that SAA induces DNA damage in HCAEC, which can be largely prevented in the presence of OLE. Furthermore, OLE decreased the susceptibility of HCAEC to oxidative DNA damage, induced by treating the cells with 3% H_2_O_2_ (Figure 4B). Less than 10% of untreated cells and none of the SAA-stimulated cells were without COMET tails (no DNA damage) upon exposure to H_2_O_2_, however the percentage of HCAEC without COMET tails increased upon treatment with OLE (Figure 4B). Furthermore, in presence of H_2_O_2_, 28% and 42% cells showed severe DNA damage (category E) in untreated and SAA-stimulated HCAEC, respectively. OLE alone or in the presence of SAA attenuated this damage (Figure 4B). Specifically, cells treated with OLE were evenly distributed in all categories, and the percentage of cells in category E was between 13 and 19% (Figure 4B). Collectively, treatment with OLE protected HCAEC from oxidative DNA damage, while the treatment of HCAEC with SAA worsened the oxidative DNA damage.

### Expression of the SAA-Induced miR-146a and Let-7e Is Altered by OLE

**Figure 5:**
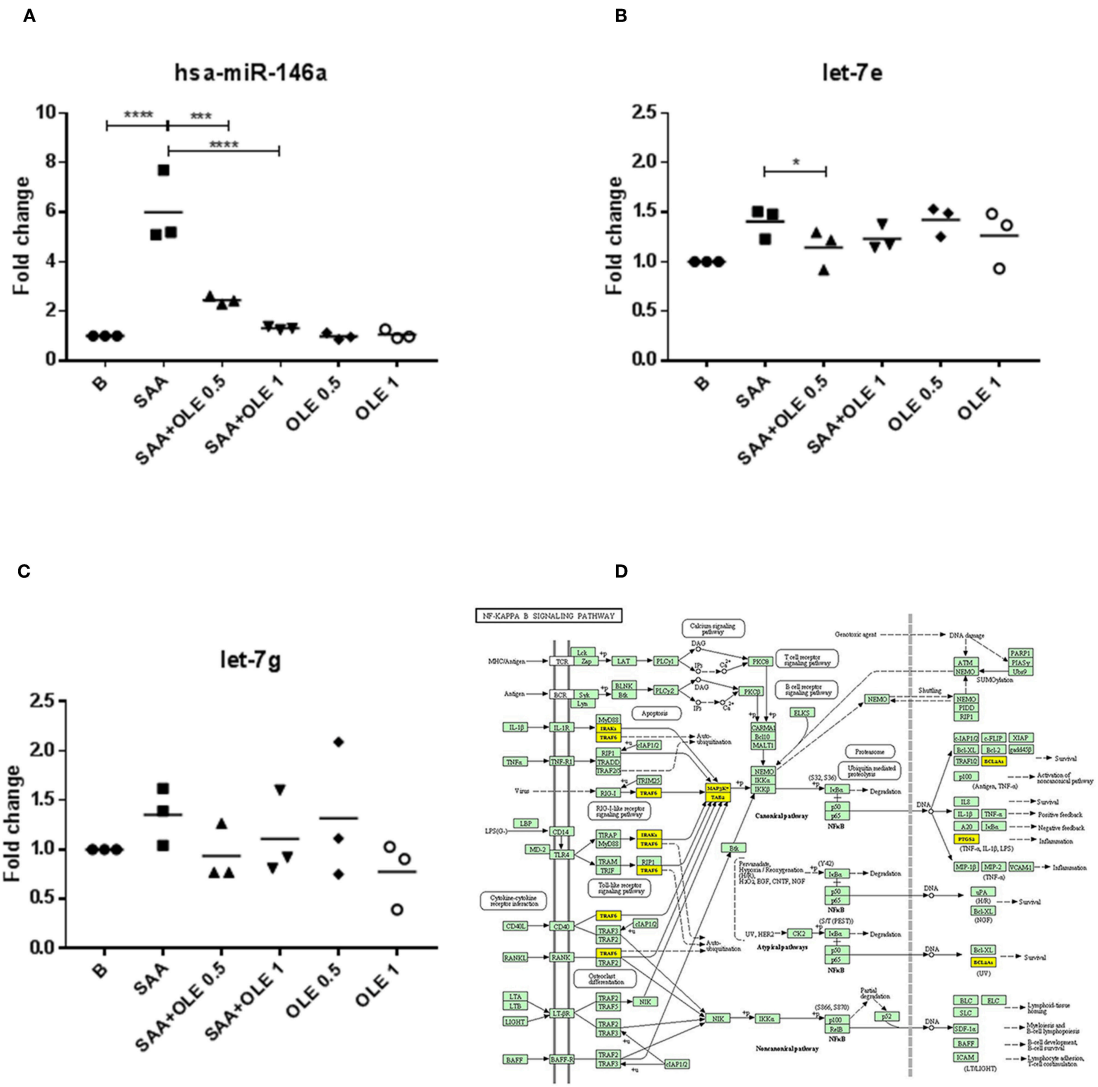
OLE regulates SAA-induced miR-146a and let-7e. **(A–C)** represent fold changes of mRNA expression levels of miR-146a, let-7e and let-7g, respectively. The mean is shown of 3 biological replicates. Data was analyzed using RM one way ANOVA test with Tukey's multiple comparison test *p*-values are shown as follows *p > 0.05, ***P < 0.001, ****P < 0.0001. **(D)** miR-146 and let-7e functional analysis showed significant influence on KEGG NF-κB signaling pathway (*p* = 0.009) (37) with 6 predicted target genes (marked yellow).

MicroRNA networks play a key role in regulating pro-inflammatory cell responses, as well as endothelial dysfunction. However, the expression and function of microRNAs during the SAA-driven endothelial activation remains largely unknown. Scarce evidence exists showing that OLE affects the microRNA profiles (35). To explore the alteration in microRNA networks during the SAA-driven endothelial activation, we measured the expression of miR-146a, let-7e, and let-7g with roles in inflammation (36) (Figures 5A–C). These measurements showed that SAA significantly increased the expression of miR-146a and OLE ameliorated the SAA-driven induction of miR-146a (Figure 5A) and let-7e (Figure 5B). The effects of SAA on the expression of let-7g (Figure 5C), however, were not changed from untreated HCAEC. Analysis of genes targeted with miR-146a and mirR-let-7e [using ≫Diana≪ tool mirPath v3 (30)] showed significant association with Toll like receptor signaling (*p* = 0.005, n of genes targeted is 9), NF-kappa B signaling (*p* = 0.009, number of genes targeted is 6, Figure 5D), ErbB signaling pathway (*p* = 0.02, number of genes targeted is 7) and Cell cycle (*p* = 0.03, number of genes targeted is 11) KEGG pathways (37).

## Discussion

Our study provides novel data on the positive effects of olive leaf extract on the human coronary artery endothelium *ex vivo*. We show that OLE decreases levels of IL-6, IL-8 released protein and E-selectin mRNA in HCAEC in response to the endogenous inflammatory SAA, likely by reducing the SAA-driven phosphorylation of NF-κB p65. Furthermore, OLE changes the SAA-driven miR-146a and let-7e expression and decreases SAA-induced and H_2_O_2_-induced oxidative DNA damage in HCAEC, indicating protection at multiple levels. Using HCAEC *in vitro* cell culture model, we mimicked OLE's actions on the endothelial cell function. However, certain effects could be elucidated further only by using *in vivo* models or interventional human studies, e.g., the effects of OLE on the cholesterol/triglyceride profiles (18, 26).

Our results are in line with the human intervention study of olive leaf supplementation, which identified NF-κB as a central biological factor influenced by OLE (27). Similarly, a dietary intervention study showed that contrary to butter and walnut meals extra virgin olive oil did not elicit the phosphorylation of NF-κB in PBMC from healthy individuals (38). Moreover, OLE inhibited the phosphorylation of NF-κB and AP-1 in HUVEC and Raw264 macrophages (24, 28). Polyphenol compounds, such as oleuropein attenuated the phosphorylation of NF-κB in mouse models of ileum ischemia/reperfusion (39) and spinal cord injury (40). Overall, this suggests that active ingredients of OLE might have a therapeutic potential in regulating the activity of NF-κB.

We show that OLE decreases the SAA-induced release of IL-6 and attenuates the SAA-driven IL-8 and pro-adhesive E-selectin in HCAEC. Moreover, OLE decreased the basal expression of both pro-adhesive molecules, VCAM-1 and E-selectin, in HCAEC. All these molecules are critical effectors in the atherosclerosis process (41–44). The attenuation of pro-inflammatory responses causes a mediator-specific effect in HCAEC, since MIF released protein levels were not affected by OLE treatment. Our results are in line with the observed decrease in serum IL-8 in different human interventional studies upon OLE consumption (25–27) and the oleuropein-driven amelioration of IL-6 production in the LPS-induced Raw264 macrophages (24). Thus, OLE could have multiple beneficial effects via reducing the pro-inflammatory responses in different cell types participating in the atherosclerotic process. Additionally, oleuropein efficiently reduced the expression of VCAM-1 and E-selectin in HUVEC upon pro-inflammatory stimulation with LPS, TNF-α and PMA (28), when compared to other phytochemicals in the Mediterranean diet (e.g., elenolic acid and tyrosol in olive oil, resveratrol in wine). Healthy volunteers and subjects with hypertriglyceridemia who consumed extra virgin olive oil had significantly lower amounts of sICAM-1 and sVCAM-1 in comparison with subjects consuming refined olive oil, which contains no polyphenols and tocopherols (45). Nevertheless, the effects of dietary olive oil and extra virgin olive oil can partially be attributed to the incorporation of oleic acid or other fatty acids in cell membranes (46).

MMPs play an integral role in the atherosclerotic process via remodeling the extracellular matrix and regulating the migration of vascular smooth muscle cells. MMP2 and MMP9 decrease plaque stability and MMP2 appears to have a particular deleterious/pro-atherogenic role (47). While SAA did not affect the expression of MMP2 and MMP9 mRNAs in our experiments, OLE decreased the expression of MMP2 in HCAEC under basal conditions. This suggests that OLE might have additional (SAA-independent) beneficial effects on the MMP2-driven atherosclerosis-associated remodeling of the vascular wall.

In addition to its anti-inflammatory and antiadhesive activities, oleuropein acts as a potent free radical scavenger that suppresses the production of reactive oxygen species in different experimental systems including H_2_O_2_-induced cell damage (19, 29). Here, we report a novel observation that SAA (at 1,000 nM) induces DNA damage in HCAEC and increases the sensitivity of HCAEC to oxidative DNA damage. These SAA-driven effects were largely prevented in the presence of OLE. Importantly, OLE also reduced the H_2_O_2_-driven oxidative damage in the absence of SAA (Figure 4). The COMET assay we have utilized in this report is increasingly used in regulatory genotoxicity testing for the evaluation of DNA damage and repair in various tissues (48). It is one of a few assays that tests for both single stranded and double stranded DNA breaks, as opposed to γ-2HAX, which detects only dsDNA breaks. The COMET assay has become a well-established and well-accepted molecular technique in human biomonitoring and in clinical studies (49, 50). Moreover, COMET is one of the measuring techniques of European Standards Committee on Oxidative DNA Damage (ESCODD) (51). There are also several advantages of using the COMET assay, namely, the assay can be calibrated to give quantitative measures (52), it is a sensitive test (53), which is cost-effective and is becoming easier to perform, with the development of the high through-put Comet-Chip (54). More recently, an EpiComet-Chip was developed and validated for the assessment of DNA methylation status (55). There appear to be opposing activities of SAA, namely substantial DNA damage induction vs. slightly increased or stable viability (56, 57). This could be due to, on the one hand, SAA inflammatory events causing DNA damage in HCAEC, while on the other hand, viability might reflect the implication of elevated SAA in multiple types of cancers (e.g., esophageal squamous cell, ovarian, breast, lung, renal, and gastric) with increasing levels correlating with severity of cancer stages (58). The question then emerges whether SAA downregulates expression of genes, such as p53, thus elevating cell survival and cell cycle progression, which could be addressed in the future.

At the microRNA level, we show that SAA increases the expression of microRNA146a and let-7e in HCAEC, while OLE significantly attenuates these SAA-induced effects. The activation of NF-κB induces the expression of microRNA-146a, which acts as a negative feedback regulator of NF-κB via targeting TRAF6 and IRAK1/2. This controls the NF-κB transcriptional activity in the presence of excessive pro-inflammatory stimulation (36). Contrary to microRNA-146a, let-7e promotes the activation of NF-κB by inhibiting the expression of IκB β in endothelial cells (59). Thus, OLE could fine-tune SAA-driven activity of NF-κB by altering cellular microRNA networks (60).

In regard to the DIANA-miRPath v3 data (30) combining target prediction algorithms, with manually curated miRNA:gene interaction datasets to chart miR-146a and let-7e targets, the following genes and their involvement in processes most likely to be affected are, among others: TLR2 pathway (TLR2, TAB2, AKT2, JUN, TRAF6, STAT1, IRF7, IRAK1, MAP3K7), NF-κB pathway (TAB2, TRAF6, PTGS2, BCL2A1, IRAK1, MAP3K7), ErbB pathway (GSK3B, EGFR, STAT5B, AKT2, JUN, CDKN1A, ABL1) and cell cycle (GSK3B, CCNB1,CDC25B, SMAD4, RBL1, CDC23, CDKN1A, PRKDC, MDM2, ABL1, CDC25A). Among these genes, several are implicated in survival (BCL2A1, EGFR, STAT5B), ubiquitin-mediated proteolysis (TAB, TRAF6, IRAK1, MAP3K7, CDC23), anti-viral effects (IRF7), metabolism (AKT2, GSK3B). Interestingly, the cell cycle genes included both promoting (e.g., CDC23, CDC25A), as well as regulating (e.g., CDKN1A, CCNB1) genes of the cell cycle. In cells in general, it would be interesting to determine whether SAA downregulates PRKDC (elevating p53) and/or upregulates MDM2 (attenuating p53). While *in silico* analysis tools and applications, such as miRPath provide support to research, *in vitro* experimentation will further be necessary to confirm specific gene involvement.

Possible mechanisms of SAA activity could include changes in gene expression of pro-inflammatory cytokines, epigenetic events (e.g., miRNA-related), as well as induced reactive oxygen, nitrogen species, which may, in turn cause damage to cellular components (e.g., DNA), leading to chronic acceleration of chronic vascular disease, with OLE providing a counterbalance to these processes. In line with this, our data suggests that OLE exhibits multiple protective actions that could prevent or attenuate pro-inflammatory activation, influence vascular remodeling via modulation of MMP2 expression and may prevent DNA damage in the coronary arterial endothelium. This suggests that olive leaf extract, or its derivatives, might have athero-protective actions also *in vivo*, which would need to be further explored in the future.

## Author Contributions

BB, KL, and MF-B designed the experiments, acquired, and analyzed the data, and wrote the manuscript. KL, BB, TK, TJ, KM-P, DT, and PŽ performed the experiments. DT, LŽ, and BS-P obtained OLE and advice on COMET. SS-S, SČ and OD coordinated the study. All authors participated in critical discussion of the data and drafting the manuscript. All authors have seen and approved the manuscript and its contents and are aware of the responsibilities connected to authorship.

### Conflict of Interest Statement

The authors declare that the research was conducted in the absence of any commercial or financial relationships that could be construed as a potential conflict of interest.
