# Supplementary material for: Olive Leaf Extract Attenuates Inflammatory Activation and DNA Damage in Human Arterial Endothelial Cells
Source: Front Cardiovasc Med. 2019 May 16;6:56. doi: 10.3389/fcvm.2019.00056 (PMC6531989; doi:10.3389/fcvm.2019.00056)
Supplement: Supplementary file 1 [file Table_1.docx]

Supplementary Material

**Supplementary Table 1.** Primers used in qPCR analysis.

| **mRNA** | **Primer sequences** |
| --- | --- |
| GAPDH | Forward 5'-TGT AGT TGA GGT CAA TGA AGG G-3'  Reverse 5'-ACA TCG CTC AGA CAC CAT G-3' |
| E-selectin | Forward 5ʼ-TCA GTG TAT CCC TCT AGT TCC C-3ʼ  Reverse 5ʼ-CTG CCA AAG CCT TGA ATC A-3ʼ |
| VCAM-1 | Forward 5ʼ-CTC AAG CAT GTC ATA TTC ACA GAA C-3ʼ  Reverse 5ʼ-AAC CCA AAC AAA GGC AGA GTA-3ʼ |
| MMP2 | Forward 5ʼ-CGA CCC ATT TAC ACC TAC ACC AA-3ʼ  Reverse 5ʼ-CAG CTC CTG AAT GCC CTT GA-3ʼ |
| MMP9 | Forward 5ʼ-CGG CAC TGA GGA ATG ATC TAA G-3ʼ  Reverse 5ʼ-CGA ACT TTG ACA GCG ACA AG-3ʼ |
